# An interpretable machine-learning model for predicting the efficacy of nonsteroidal anti-inflammatory drugs for closing hemodynamically significant patent ductus arteriosus in preterm infants

**DOI:** 10.3389/fped.2023.1097950

**Published:** 2023-04-04

**Authors:** Tai-Xiang Liu, Jin-Xin Zheng, Zheng Chen, Zi-Chen Zhang, Dan Li, Li-Ping Shi

**Affiliations:** ^1^ Department of NICU, The Children's Hospital, Zhejiang University School of Medicine, National Clinical Research Center for Child Health, Hangzhou, China; ^2^ Department of Nephrology, Ruijin Hospital, Institute of Nephrology, Shanghai Jiao Tong University School of Medicine, Shanghai, China; ^3^ School of Global Health, Chinese Center for Tropical Diseases Research, Shanghai Jiao Tong University School of Medicine, Shanghai, China; ^4^ Yiwu Branch, Children's Hospital Zhejiang University School of Medicine, Yiwu, China

**Keywords:** patent ductus arteriosus, nonsteroidal anti-inflammatory drugs, interpretable machine learning, preterm infant, predictive model

## Abstract

**Background:**

Nonsteroidal anti-inflammatory drugs (NSAIDs) have been widely used in the closure of ductus arteriosus in premature infants. We aimed to develop and validate an interpretable machine-learning model for predicting the efficacy of NSAIDs for closing hemodynamically significant patent ductus arteriosus (hsPDA) in preterm infants.

**Methods:**

We assessed 182 preterm infants ≤ 30 weeks of gestational age first treated with NSAIDs to close hsPDA. According to the treatment outcome, patients were divided into a “success” group and “failure” group. Variables for analysis were demographic features, clinical features, as well as laboratory and echocardiographic parameters within 72 h before medication use. We developed the machine-learning model using random forests. Model performance was assessed by the area under the receiver operating characteristic curve (AUC). Variable-importance and marginal-effect plots were constructed to explain the predictive model. The model was validated using an external cohort of two preterm infants who received ibuprofen (p.o.) to treat hsPDA.

**Results:**

Eighty-three cases (45.6%) were in the success group and 99 (54.4%) in the failure group. Infants in the success group were associated with maternal chorioamnionitis (*p* = 0.002), multiple births (*p* = 0.007), gestational age at birth (*p* = 0.020), use of indometacin (*p* = 0.007), use of inotropic agents (*p* < 0.001), noninvasive ventilation (*p* = 0.001), plasma albumin level (*p* < 0.001), PDA size (*p* = 0.038) and Vmax (*p* = 0.013). Multivariable binary logistic regression analysis showed that maternal chorioamnionitis, multiple births, use of indomethacin, use of inotropic agents, plasma albumin level, and PDA size were independent risk factors influencing the efficacy of NSAIDs (*p* < 0.05). The AUC of the random forest model was 0.792. The top-three features contributing most to the model in the variable-importance plot were the plasma albumin level and platelet count 72 h before treatment and 24-h urine volume before treatment. In the external cohort, treatment succeeded in one case and failed in the other. The probabilities of success and failure predicted by the random forest model were 60.2% and 48.4%, respectively.

**Conclusion:**

Based on clinical, laboratory, and echocardiographic features before first-time NSAIDs treatment, we constructed an interpretable machine-learning model, which has a certain reference value for predicting the closure of hsPDA in premature infants under 30 weeks of gestational age.

## Introduction

Patent ductus arteriosus (PDA) is a condition in which the ductus arteriosus fails to close after birth. Hemodynamically significant patent ductus arteriosus (hsPDA) is a common complication in preterm infants, and its incidence is associated mainly with gestational age and birthweight (1). Persistent hsPDA (through which a continuous large right-to-left shunt occurs) can cause congestion of the pulmonary circulation and ischemia in the systemic circulation, thereby leading to dysfunction of multiple organs and increasing the risk of death (2).

Nonsteroidal anti-inflammatory drugs (NSAIDs), particularly ibuprofen and indomethacin, are first-line treatment to close hsPDA for preterm infants (3). The main mechanism of action of NSAIDs is to inhibit the active site of cyclooxygenase to reduce prostaglandin synthesis, thereby promoting constriction or closure of the ductus arteriosus (4). However, the ductal smooth muscle is immature in preterm infants. Hence, they may respond poorly to drugs, and have a risk of failure of PDA closure that requires a second type of treatment (medication or surgery). Various factors have been shown to influence the efficacy of NSAIDs for closing PDA, such as maternal-health status (5), gestational age (6), age in days (7), platelet count (8), and the type and dose of drugs (9). To reveal variables with nonlinear and complex relationships with the treatment outcome of NSAIDs in premature infants with hsPDA, an effective method to develop accurate predictive models is needed.

“Machine learning” is an important branch of artificial intelligence. It has been used widely in biomedicine and medicine, such as clinical diagnosis, precise treatment, and health monitoring. Machine learning involves learning and utilizing patterns in given data using advanced algorithms for making decisions or predictions about real-world events (10). However, models of machine learning are most often “black boxes” that cannot be directly interpretable to humans. Lack of interpretability undermines clinicians' trust in these black-box models, but also limits their operability for clinical prediction (11). Interpretable machine learning can provide understandable explanations for how a model works and why it makes specific predictions. It can help “debugging” of a model, direct collection of future data, provide reliable information for feature construction and human decision-making and, eventually, build trust between humans and models (12).

Here, we assessed 182 infants receiving NSAIDs for the first time to close PDA to develop an interpretable model of machine learning. This model aimed to predict the efficacy of NSAIDs for treating hsPDA in premature infants, and explain the relationship between clinical features and the treatment outcome. We hope this model can provide guidance for the clinical treatment of premature infants with PDA.

## Materials and methods

With the approval of the Institutional Review Board (IRB No. 2022-IRB-194), we evaluated preterm infants (gestational age ≤30 weeks) who were first treated with NSAIDs to close PDA at two tertiary medical centers in different regions of Zhejiang Province, China. Patients were excluded if they had one of the following conditions: (i) spontaneous closure of PDA after hospital admission; (ii) drug contraindications that necessitated direct surgical ligation; (iii) a history of NSAIDs treatment in other hospitals; (iv) complex congenital heart disease except PDA, atrial septal defect or small ventricular septal defect; and (v) incomplete data. All infants were treated with ibuprofen suspension or indomethacin.

**Figure 1:**
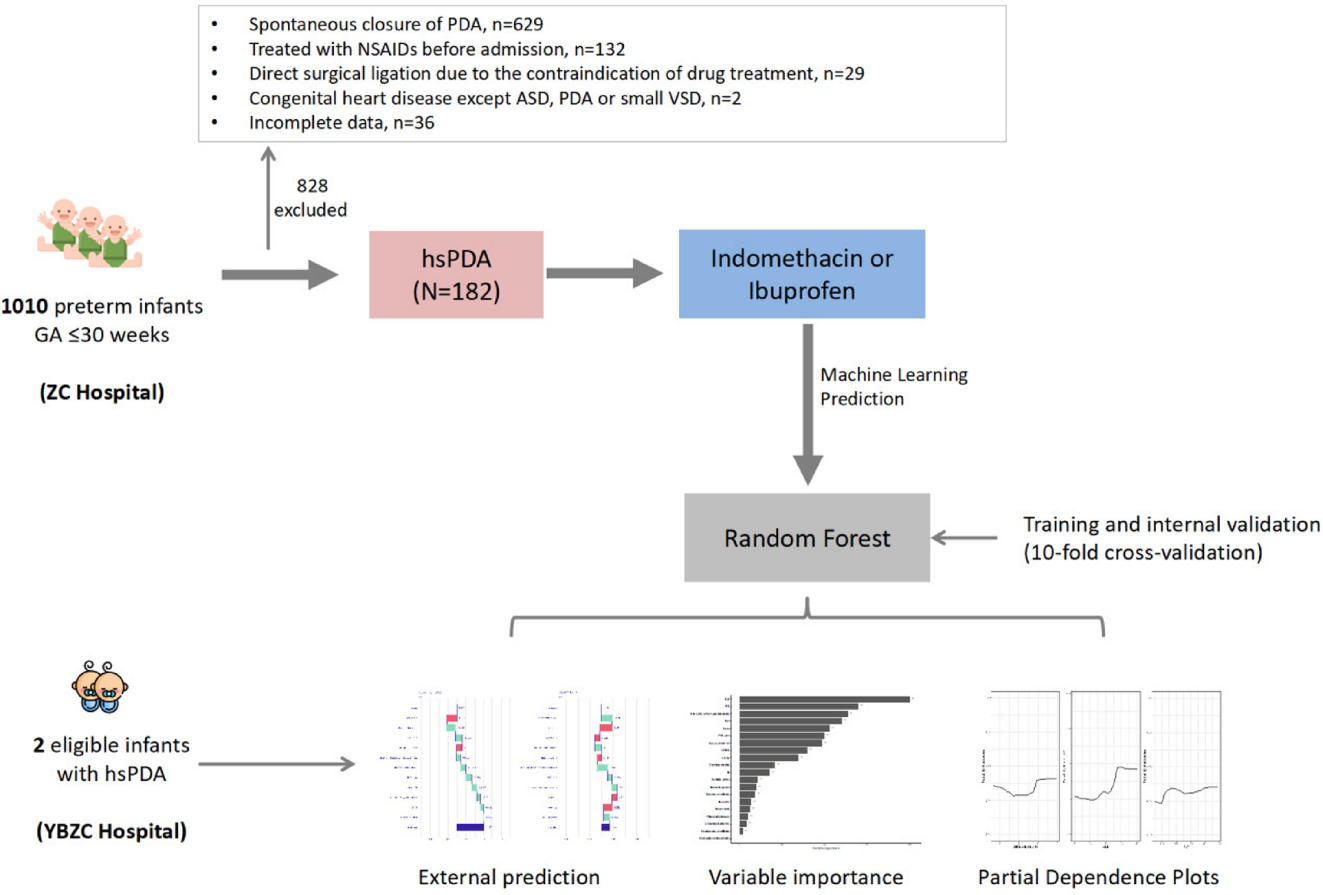
Graphic abstract. GA, gestational age; PDA, patent ductus arteriosus; ASD, atrial septal defect; VSD, ventricular septal defect; hsPDA, haemodynamically significant patent ductus arteriosus.

Patients treated at ZCH between August 2015 and July 2022 were set as the “development cohort”. Two patients treated at Yiwu Branch Of Children's Hospital Zhejiang University School Of Medicine (YBZCH) in July 2022 were set as the “validation cohort”. The graphic abstract is shown as Figure 1.

### Data collection

We collected, from medical records, maternal information (gestational hypertension, gestational diabetes mellitus, chorioamnionitis, multiple births, cesarean section, placental abruption, placenta previa, prenatal therapy of corticoid), neonatal information (gestational age, birthweight, sex, small-for-gestational-age, Apgar score at 1 min and 5 min, use of alveolar surfactants), neonatal conditions at the time of treatment (age in days, bodyweight, type of NSAID, oxygen support before treatment, use of positive inotropic drugs and the score before treatment, 24-h urine volume before the first dose of treatment, gastrointestinal complications (including bleeding and perforation during treatment)), laboratory results within 72 h before treatment (platelet count, hematocrit, level of C-reactive protein, plasma level of albumin), and echocardiographic parameters within 72 h before treatment (PDA size, maximum systolic velocity across the ductus arteriosus (V_max_), left atrial-to-aortic-root diameter ratio (LA:Ao), left ventricular end-diastolic diameter (LVEDD), left ventricular ejection fraction(LVEF)).

The definition of hsPDA was: PDA diameter ≥1.5 mm, LA:Ao ≥1.4, end-diastolic aortic regurgitation, and the clinical manifestations of systemic ischemia and pulmonary congestion (e.g., continuous murmurs, tachycardia, hypotension, increased pulse pressure, oliguria, apnea, pneumorrhagia, increased need for oxygen and respiratory support) (13). Preterm infants received an oral suspension of ibuprofen or indomethacin to treat hsPDA. Ibuprofen was administered at 10 mg/kg, 5 mg/kg, and 5 mg/kg for the first, second, and third doses, respectively, once every 24 h, for a total of three times. Indomethacin was given once every 12 h for three times in total, after being dissolved fully in 95% ethanol and diluted with warm water. The indomethacin dose was dependent upon the infant's age at the time of drug use: 0.1 mg/kg per dose for infants <48 h after birth, 0.2 mg/kg per dose for infants >48 h but <7 days after birth, and 0.25 mg/kg per dose for infants >7 days after birth. If the urine volume was >1 ml/kg·h after one dose, the next dose could be given at an interval of 12 h; if the urine volume was 0.6–1 ml/kg·h, the interval should be 24 h; if oliguria (<0.6 ml/kg·h) or anuria occurred, we discontinued the drug and administered a low dose of dopamine (2–3 µg/kg·min). If severe gastrointestinal bleeding or perforation occurred, the drug was discontinued. After hospital admission, all preterm infants were monitored with echocardiography at least once a week until PDA was closed. Successful closure of hsPDA was considered if echocardiography confirmed PDA closure or if non-significant hemodynamics 72 h after treatment were documented.

### Development and validation of a model of machine learning

The dataset was divided randomly into a “training set” (90%) and a “test set” (10%). A predictive model of machine learning was built based on the training set using 10-fold cross-validation. The predictive performance of the model was assessed using the test set. The prediction accuracy was evaluated by calculating the area under the receiver operating characteristic curve (AUC). The trained model was validated using external cohort data to achieve “individualized” prediction.

### “Explainability” of the model

Models of machine learning can provide accurate predictions, but lack sufficient interpretability. In clinical decision-making, interpreting predictive models of machine learning correctly—opening the black box—is important for healthcare workers to understand the source of results. Interpretable machine learning can improve the transparency and traceability of the decision-making process.

We constructed an interpretable machine-learning algorithm based on random forests. Variables were selected using the method of recursive-feature elimination to determine the final predictor variables entered in the model. After ranking of “variable importance”, we further explored the relationship between independent variables and dependent variables. According to the importance of variables, the marginal effects of the top-three independent variables on the dependent variable were plotted to reveal how y (probability of success) changed with the independent variable x, thereby realizing interpretive analysis of the machine-learning model.

### Statistical analyses

Numerical data with a normal distribution are described as the mean ± standard deviation, and were compared using the Student's t-test. Numerical data with a non-normal distribution are described as the median (quartile 1, quartile 3), and were compared using the nonparametric test. Categorical data are described as the frequency (percentage), and were compared using the chi-square test. Binary logistic regression analysis was used to determine factors affecting treatment efficacy, and variables with *p* < 0.05 were included in a multivariable logistic regression model for forward selection. The random forest model was built using the “caret” package, and the significance of importance metrics for the model was estimated using the “rfPermute” package. Partial-dependence plots were constructed using the “pdp” package. Statistical analyses were undertaken using R 3.6.3 (R Institute for Statistical Computing, Vienna, Austria). *p* < 0.05 was considered significant.

## Results

### General data

Between August 2015 and July 2022, we reviewed the medical records of 182 preterm infants of gestational age ≤30 weeks who were first treated with NSAIDs to close hsPDA. Ninety-two cases (50.5%) received indomethacin, and 90 (49.5%) received ibuprofen. Eighty-three patients (45.6%) had successful closure of PDA, whereas 99 (54.4%) required secondary treatment (medication or surgical ligation) (Figure 1). The demographic data of preterm infants in the group that had treatment success and the group that suffered treatment failure are shown in Table 1. There were significant differences between the two groups in terms of: chorioamnionitis; multiple pregnancy; gestational age; use of indomethacin; use of inotropic agents; invasive or noninvasive ventilation; plasma albumin level, PDA size; V_max_. The remaining variables showed no significant differences between the two groups. Multivariable binary logistic regression analysis showed that maternal chorioamnionitis, multiple births, use of indomethacin, use of inotropic agents, plasma albumin level, and PDA size were independent risk factors influencing the efficacy of NSAIDs (Table 2).

**Table 1:** Demographic characteristics.

Variables	Success group (*n *= 83)	Failure group (*n *= 99)	T/Z/*χ*^2^	*p-*value
**Maternal factors**				
Gestational hypertension, *n* (%)	12 (14.5)	16 (16.2)	0.101	0.751
GDM, *n* (%)	19 (22.9)	20 (20.2)	0.194	0.660
Chorioamnionitis, *n* (%)	34 (41)	63 (63.6)	9.324	0.002
Placental diseases, *n* (%)	16 (19.3)	13 (13.1)	1.273	0.259
Cervical incompetence, *n* (%)	7 (8.4)	16 (16.2)	2.442	0.118
Thyroid diseases, *n* (%)	8 (9.6)	10 (10.1)	0.011	0.917
ICP, *n* (%)	2 (2.4)	1 (1)		0.593
PCOS, *n* (%)	2 (2.4)	4 (4)		0.690
Cesarean section, *n* (%)	30 (36.1)	32 (32.3)	0.294	0.588
Prenatal therapy of corticoid, *n* (%)	14 (16.9)	24 (24.2)	1.486	0.223
Multiple births, *n* (%)	24 (28.9)	48 (48.5)	7.231	0.007
**Neonatal factors**				
Gestational age(wk), mean (SD)	27.7 (26.4–28.9)	27 (26–28.1)	−2.325	0.020
Birthweight (g), median (IQR)	1,020 (860–1190)	940 (830–1125)	−1.732	0.083
Male gender, *n* (%)	45 (54.2)	58 (58.6)	0.351	0.554
SGA, *n* (%)	3 (3.6)	3 (3)		0.572
Apgar score at 1 min, mean (SD)	6.7 ± 2.6	6.5 ± 2.8	0.345	0.730
Apgar score at 5 min, mean (SD)	8.2 ± 2.0	8.4 ± 1.9	−0.682	0.496
Surfactant therapy, *n* (%)	66 (79.5)	81 (81.8)	0.154	0.695
**Treatment factors**				
Age at treatment (day), mean (SD)	11.2 ± 6.6	10 ± 6.3	1.319	0.189
Weight at treatment (g), mean (SD)	1080 ± 274	1038 ± 302	0.966	0.335
24-h urine volume before administration (ml/kg.h), median (IQR)	3.6 (3.2–4.3)	3.7 (3–4.5)	−0.541	0.588
Indometacin, *n* (%)	51 (61.4)	41 (41.4)	7.247	0.007
Ibuprofen, *n* (%)	32 (38.6)	58 (58.6)	7.247	0.007
Dopamine, *n* (%)	25 (30.1)	30 (30.3)	0.001	0.979
Inotropic agents, *n* (%)	11 (13.3)	37 (37.4)	13.528	<0.001
IS, mean (SD)	1.6 ± 6.4	2.8 ± 5.2	−1.306	0.193
Invasive ventilation, *n* (%)	26 (31.3)	56 (56.6)	11.619	0.001
Noninvasive ventilation, *n* (%)	50 (60.2)	39 (39.4)	7.852	0.005
Nasal cannula for oxygen, *n* (%)	7 (8.4)	5 (5.1)	0.839	0.360
Gastrointestinal symptoms, *n* (%)	18 (21.7)	18 (18.2)	0.350	0.554
Infection during medication, *n* (%)	9 (10.8)	8 (8.1)	0.407	0.524
**Laboratory and echocardiographic factors**				
PLT (×10^9^/L), mean (SD)	208 ± 92	183 ± 86	1.881	0.062
Hct (%), median (IQR)	39.5 (35.1–44.9)	39.7 (35.1–42.9)	−0.459	0.646
ALB (g/L), median (IQR)	30.1 (28–33.1)	27.6 (26–30.6)	−3.851	<0.001
PDA size (mm), mean (SD)	2.9 ± 0.6	3.1 ± 0.6	−2.093	0.038
LA:Ao, mean (SD)	1.2 ± 0.2	1.2 ± 0.2	1.425	0.156
LVEDD (mm), mean (SD)	14.4 ± 2.1	14.1 ± 2.4	0.795	0.428
Vmax (m/s), mean (SD)	2.1 ± 0.8	1.8 ± 0.6	2.506	0.013
LVEF (%), median (IQR)	69 (63–72)	68 (64–72)	−0.528	0.598

GDM, gestational diabetes; ICP, intrahepatic cholestasis; PCOS, polycystic ovary syndrome; SGA, small for gestational age; IS, inotropics score; PLT, platelet; Hct, hematocrit; ALB, albumin; PDA, patent ductus arteriosus; LA/Ao, left atrial-to-aortic-root diameter ratio; LVEDD, left ventricular end-diastolic diameter; Vmax, maximum systolic velocity across the ductus arteriosus; LVEF, left ventricular ejection fraction. Data expressed as mean (SD) or median (25th; 75th percentile) or as number (%).

**Table 2:** Multivariate logistic regression analysis of risk factors of drug treatment effectiveness.

	B	SE	Wals	*P*	OR	95%CI
Multiple births	−0.742	0.355	4.366	0.037	0.476	0.238–0.955
Chorioamnionitis	−0.984	0.354	7.721	0.005	0.374	0.187–0.748
Indometacin	0.739	0.363	4.157	0.041	2.094	1.029–4.262
Inotropic agents	−1.078	0.447	5.824	0.016	0.340	0.142–0.817
ALB	0.121	0.051	5.614	0.018	1.128	1.021–1.247
PDA size	−0.748	0.300	6.220	0.013	0.473	0.263–0.852
Constant	−0.784	1.763	0.198	0.657	0.457	

ALB, albumin; PDA, patent ductus arteriosus.

### Model prediction using random forests

**Figure 2:**
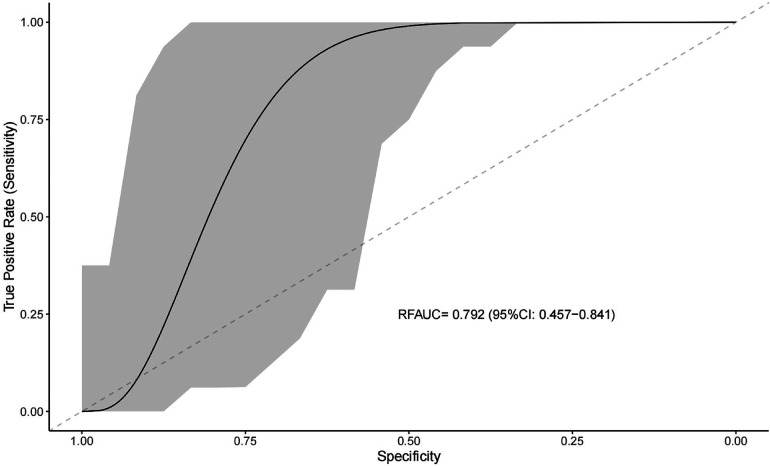
Receiver operating characteristic curve for evaluating the predictive ability of the machine learning system.

We used clinical features to predict the efficacy of NSAIDs for PDA closure. When all variables were entered into the equation, the model showed high prediction accuracy. Therefore, all factors were introduced to build the predictive model in the training set, followed by assessment of prediction accuracy using the test set. The AUC of prediction by the model was 0.792 (95%CI: 0.457–0.841) (Figure 2).

### Variable importance and marginal effect

**Figure 3:**
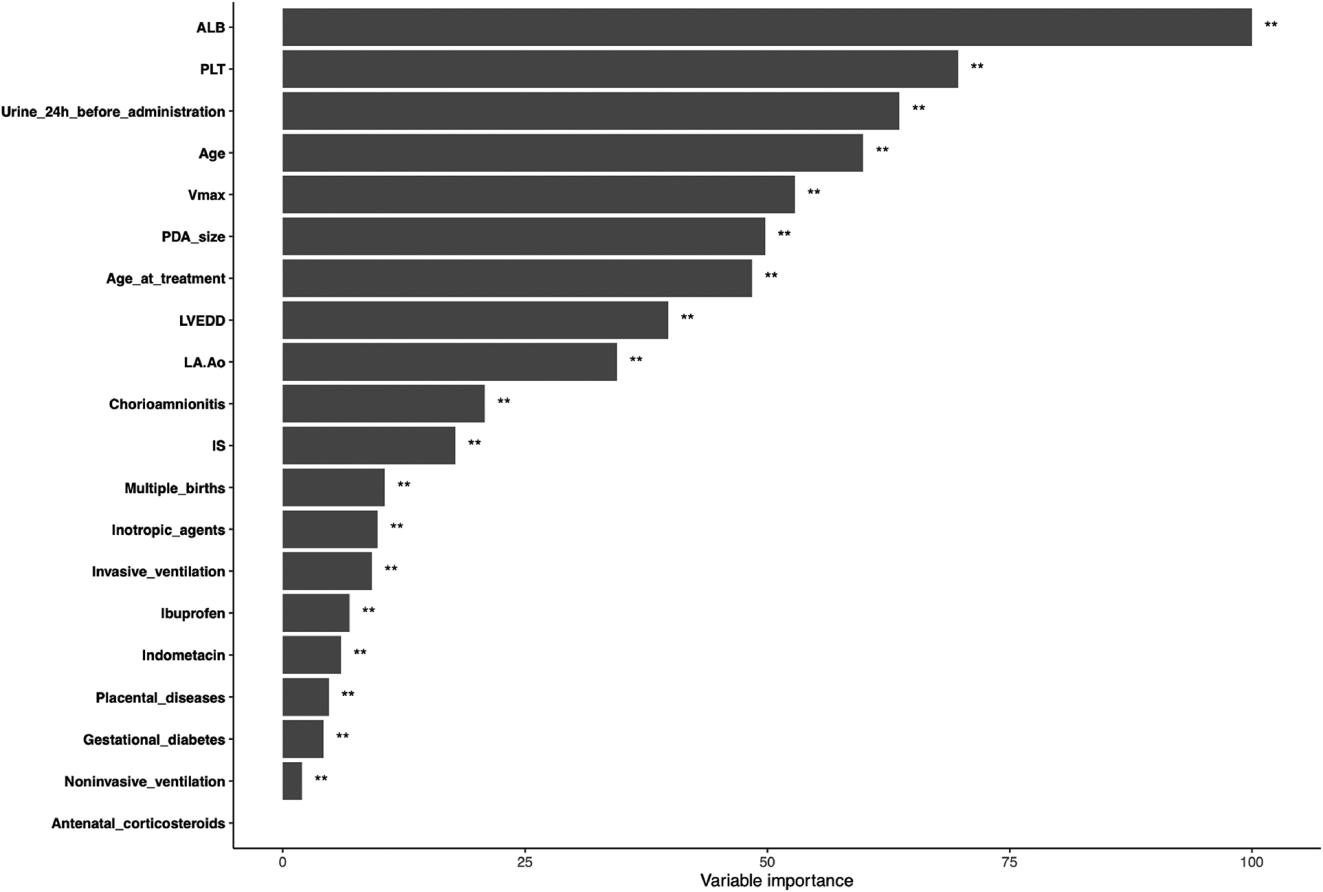
Variable importance of proposed model. ALB, albumin; PLT, platelet; Age, gestational age; Vmax, maximum systolic velocity across the ductus arteriosus; PDA, patent ductus arteriosus; LVEDD, left ventricular end-diastolic diameter; LA:Ao, left atrial-to-aortic-root diameter ratio; IS, inotropics score.

**Figure 4:**
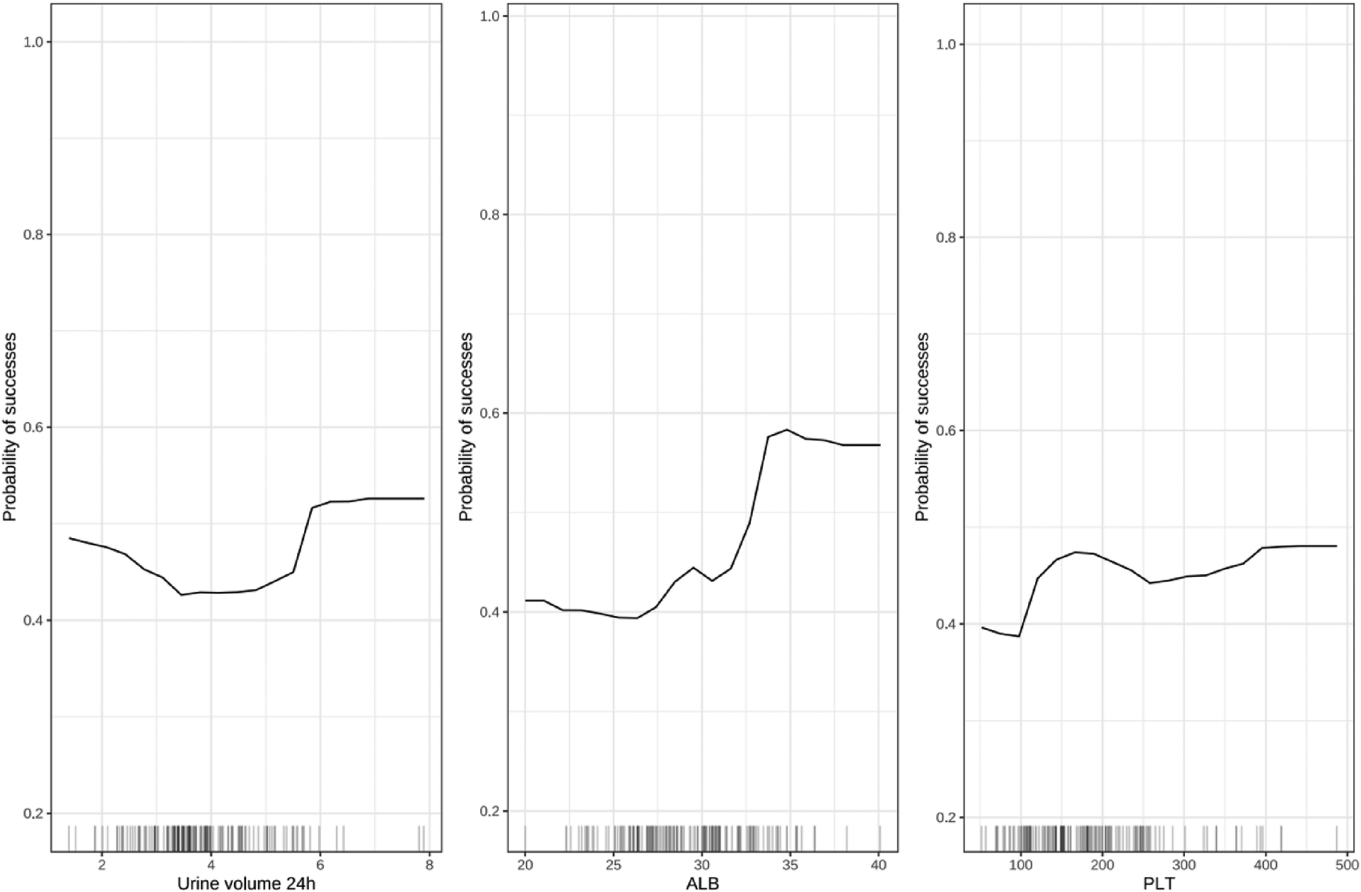
Marginal effect diagram of independent variable (top-three) and dependent variable. ALB, albumin; PLT, platelet.

To overcome the lack of interpretability of machine learning (i.e., understanding the degree of contribution of independent variables to model prediction), we used contribution score rankings to determine the reliability of predictor variables. The plot for variable importance for the random forest model is shown in Figure 3. The five most important variables affecting the model were: pre-treatment plasma albumin level; platelet count; 24-h urine volume; gestational age; V_max_. All of these variables were significant. To further identify the factors contributing most to the predictive model, we selected the top-three independent variables according to importance to plot the marginal effects of independent variables on the dependent variable. Nonlinear relationships were observed between the pre-treatment albumin level, platelet count, and 24-h urine volume and treatment outcome (successful PDA closure) (Figure 4) (Marginal effect plots of other variables are seen in Supplementary Materials S1, S2). If the urine volume was 3.5–5.5 ml/kg·h, albumin level was <27 g/L, and platelet count was <100 × 10^9^/L, then the probability of successful closure of hsPDA with NSAIDs administration was low. The probability of success increased with an increase in the albumin level (within 27–34 g/L) and platelet count (within 100–175 × 10^9^/L). However, if the plasma albumin level and platelet count continued to increase, the probability of success did not increase further.

### Validation using an external cohort

**Figure 5:**
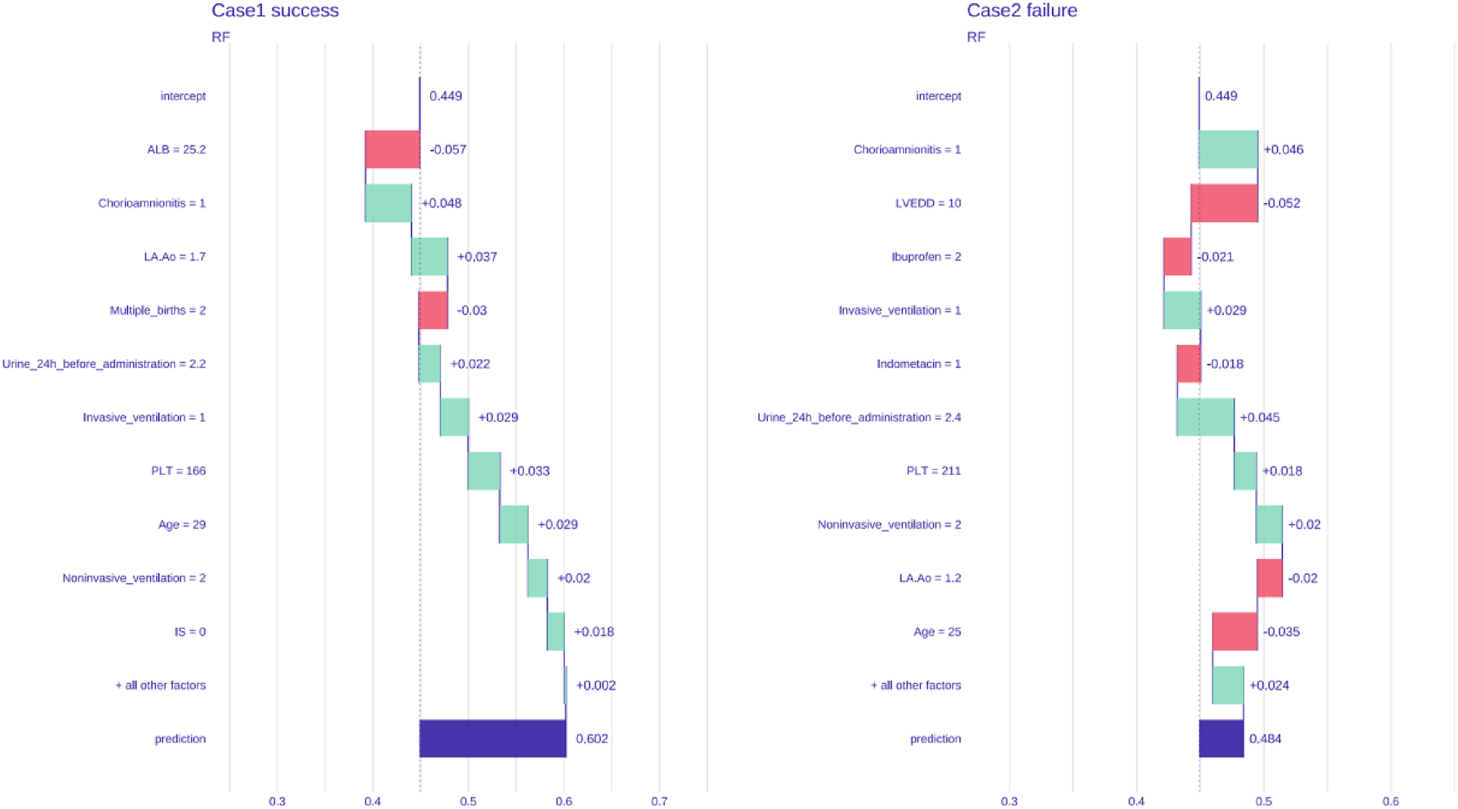
External prediction of treatment outcome of two premature infants.

The model was validated using the external data of two preterm infants who received ibuprofen (p.o.) to close hsPDA at another medical center. One patient was treated successfully, whereas the other patient was not. The probabilities of success and failure predicted by the model were 60.2% and 48.4%, respectively (Figure 5).

## Discussion

In this retrospective cohort study, we developed and validated a machine-learning algorithm that used 11 maternal features, 20 neonatal features, and 8 laboratory and echocardiographic features to predict the efficacy of first-time treatment with NSAIDs to close hsPDA in preterm infants. The random forest model demonstrated good predictive performance and clinical interpretability. Among the top-10 features in the plot for variable importance, over half were laboratory and echocardiographic features. We illustrated how the top-three important features affected the treatment outcome using a marginal effect plot. Furthermore, we validated this predictive model with two external cases from another medical center. All the features used in the model are routine clinical variables collected before medication use. Therefore, the model can be helpful in clinical decision-making for preterm infants in the neonatal intensive care unit, which demonstrates the potential clinical value of the model.

NSAIDs have been used widely as alternatives to surgical PDA ligation for preterm infants. However, 10%–40% of patients obtain no benefits from such drugs (5). Some cohort studies or studies using regression analysis have discovered various maternal and neonatal clinical features that could be used to predict the likelihood of PDA closure with NSAIDs, such as gestational hypertension, gestational age, twin pregnancy, respiratory distress syndrome, age in days, and prenatal exposure to drugs such as magnesium sulfate and NSAIDs (5, 7, 14–17). Changes in urine volume or pulse pressure before and after NSAIDs treatment are not useful predictors of the responsiveness of preterm infants with PDA to a drug (18, 19). Those findings provide a reference for early recognition of the outcome of medication therapy and subsequent individualized management strategies for PDA. In addition, bedside ultrasonography can be used to assess the hemodynamic changes associated with the development, progression, and treatment of hsPDA (20, 21). Accordingly, we developed a machine-learning system to predict the probability of successful closure of PDA based on clinical features as well as laboratory and echocardiographic parameters 72 h before treatment. Our system demonstrated a reliability to predict the efficacy of NSAIDs for PDA closure in preterm infants of gestational age ≤30 weeks.

We wished to minimize the black-box effect and increase the transparency of our model. Hence, we interpreted the variable importance of the predictive model using plots for variable importance and marginal effect. According to these plots, the plasma albumin level and platelet count within 72 h before treatment and 24-h urine volume before treatment affected the therapeutic effect significantly. These exploratory findings may improve understanding of the features linked with the success or failure of NSAIDs therapy for preterm infants with PDA. Our findings may be useful for formulating strategies to reduce the risk of treatment failure in the future.

The relationship between the plasma albumin level and efficacy of NSAIDs for PDA closure has been reported rarely. NSAIDs have high affinity with plasma proteins (especially albumin) and bind to them in a reversible manner, which can affect the dose–response relationship and the therapeutic effect of drug combinations (22). With regard to hypoproteinemia, the free drug concentration in plasma increases, which can enhance drug efficacy and even cause adverse reactions but, simultaneously, alter the half-life and duration of action of the drug. We found that the efficacy of NSAIDs for PDA closure improved with an increase in the plasma albumin level within a certain range, further indicating that the efficacy of the drug may be dependent upon its duration of action.

Studies have demonstrated the association between the platelet count and response to a drug in treatment of hsPDA. A low platelet count is associated with a higher possibility of failure of PDA treatment using ibuprofen or indomethacin, whereas a high platelet count (≥181 × 10^9^/L) independently increases the probability of success of treatment with ibuprofen (8, 23). However, a recent study showed that increasing the platelet count to relatively high levels by transfusion to correct thrombocytopenia did not aid PDA closure, and instead increased the risk of intraventricular hemorrhage (24). Those contradictory results could be explained by our marginal-effect plot of model variables. The probability of success with NSAIDs was low when the platelet count <100 × 10^9^/L, and increased correspondingly if the platelet count was within 100–175 × 10^9^/L; but if platelet count continued to increase, the probability of treatment success did not increase further. Further research is needed to elucidate the mechanism of PDA closure and explore the potential value of platelet transfusion for improving PDA closure using medications.

Indomethacin and ibuprofen are nonselective inhibitors of cyclooxygenase. They can cause renal vasoconstriction, reduced renal blood flow, and acute kidney injury. NSAIDs-associated acute kidney injury can manifest as reduced urine volume and/or increased serum levels of urea/creatinine (25, 26). Therefore, reduced urine volume is considered to be a potential predictor of the efficacy of NSAIDs for closing PDA. We revealed that the 24-h urine volume before treatment was an important variable affecting the treatment outcome of NSAIDs. The probability of treatment success was low if the urine volume was 3.5–5.5 ml/kg·h. One study reporting on the relationship between urine-volume changes and PDA closure after indomethacin treatment in preterm infants concluded that urine-volume changes could not be used to predict therapeutic effects. Therefore, whether changes in urine volume can predict the therapeutic effects of NSAIDs merits further investigation (18).

## Limitations

Our study had several limitations. First, the model is restrospective and focused only on preterm infants who received NSAIDs treatment regimens for the first time to close hsPDA, not including with those in the literature described opportunities of escalation i.e., like increasing the dose of ibuprufene or shortening of the time intervall for indomethacine. Second, plasma albumine is not very common as a parameter in laboratory diagnostics today, especially in times, where less blood samples are made in premature infants. Finally, the sample size for external validation was extremely small due to the small number of premature infants admitted in another center and the incomplete data, which may have influenced the performance of the predictive machine-learning model. Large-sample prospective studies are needed to investigate the application of interpretable machine learning-based predictive models for improving the treatment outcome of preterm infants with hsPDA in clinical practice.

## Conclusion

Based on clinical, laboratory, and echocardiographic features before first-time NSAIDs treatment, we constructed an interpretable machine-learning model, which has a certain reference value for predicting the closure of hsPDA in premature infants under 30 weeks of gestational age.

## Data Availability

The original contributions presented in the study are included in the article/**Supplementary Material**, further inquiries can be directed to the corresponding author/s.
