# Supplementary figures and images for: An interpretable machine-learning model for predicting the efficacy of nonsteroidal anti-inflammatory drugs for closing hemodynamically significant patent ductus arteriosus in preterm infants
Source: Front Pediatr. 2023 Apr 4;11:1097950. doi: 10.3389/fped.2023.1097950 (PMC10110971; doi:10.3389/fped.2023.1097950)

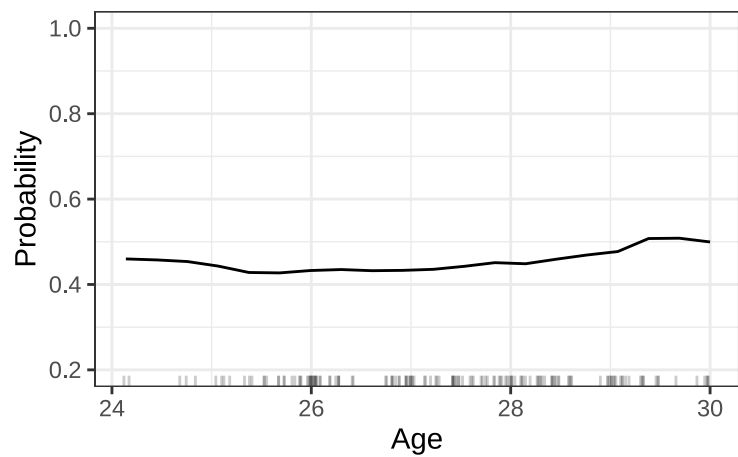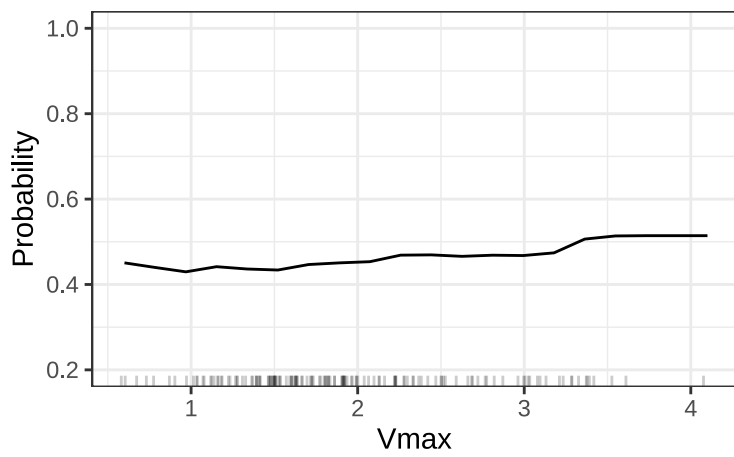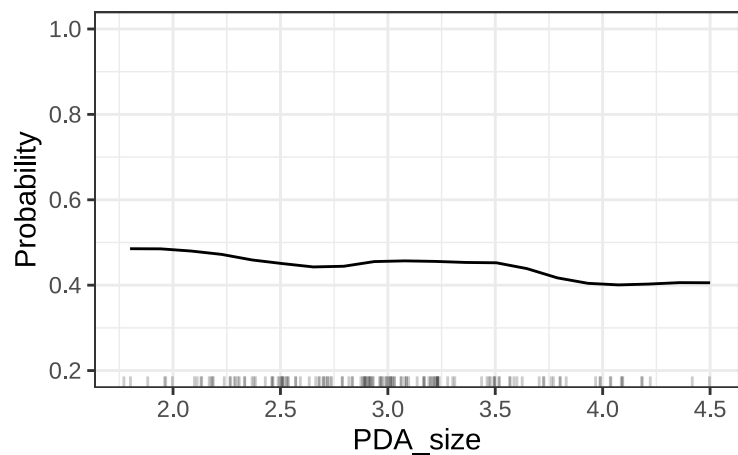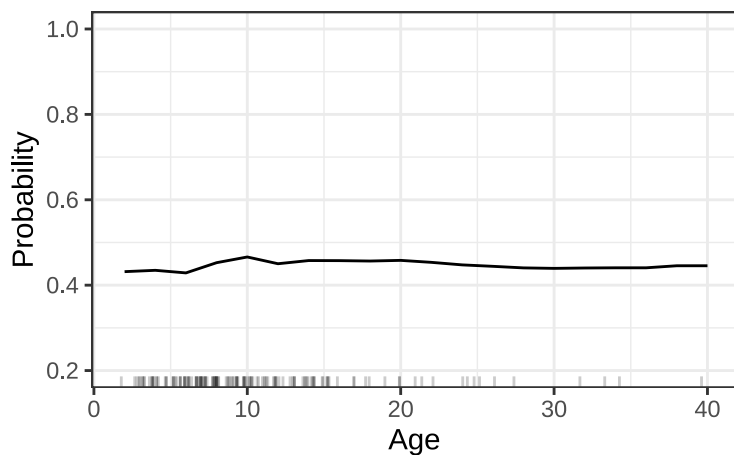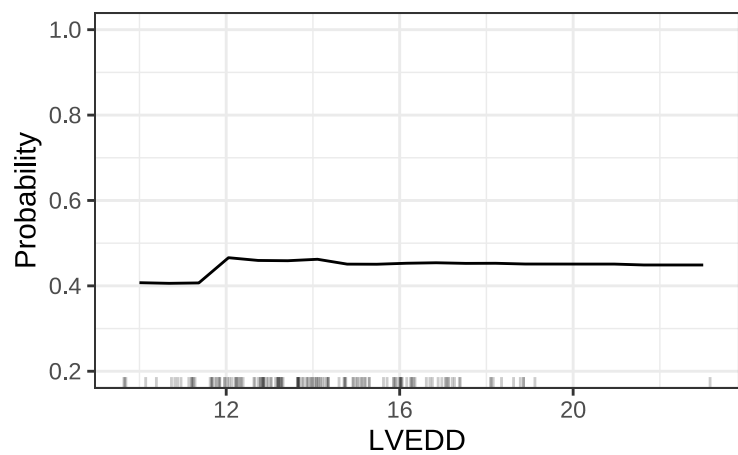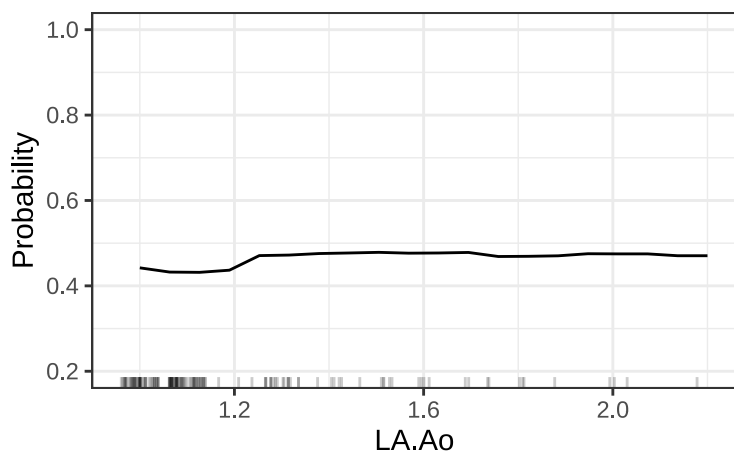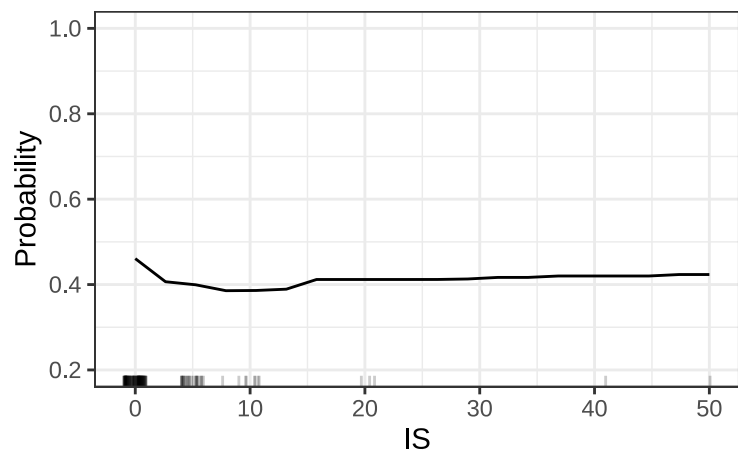

Supplement: Supplementary file 1 [file Datasheet1.pdf]

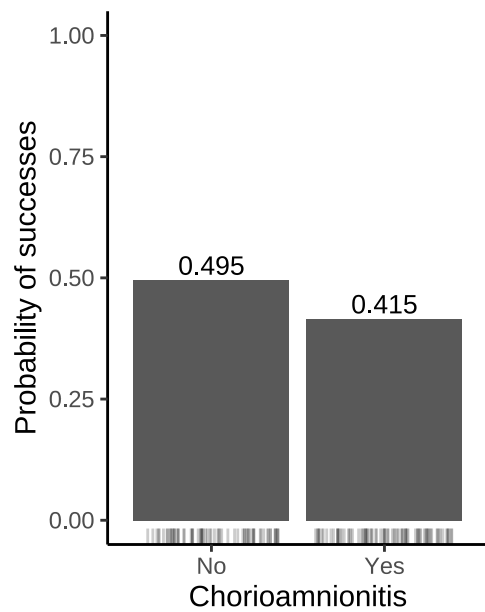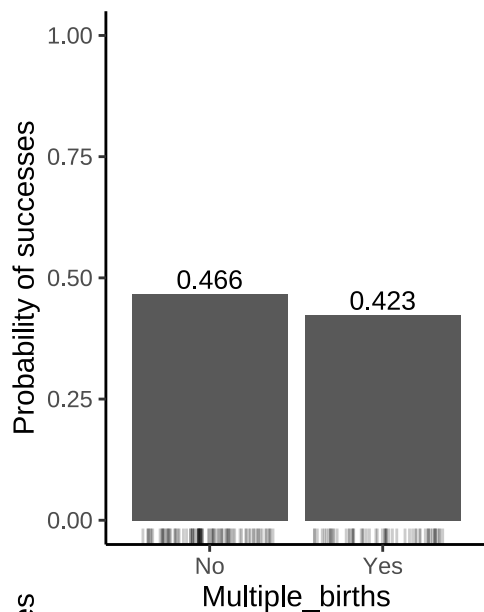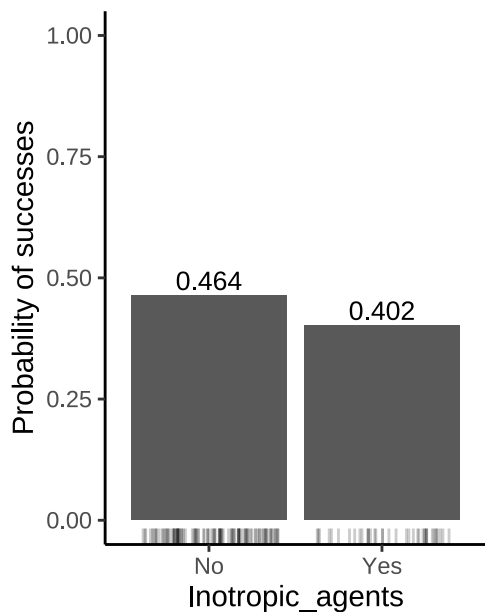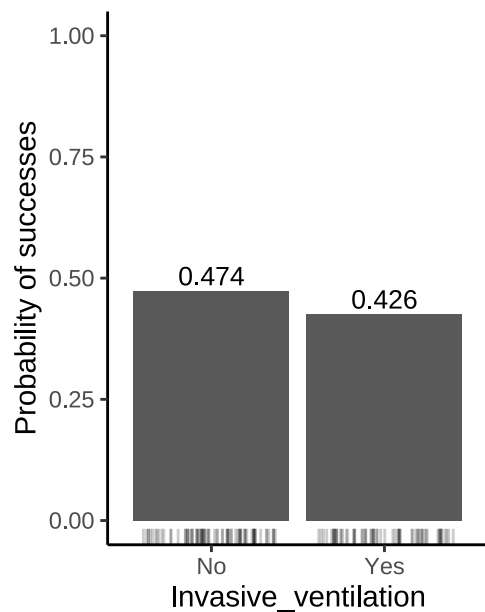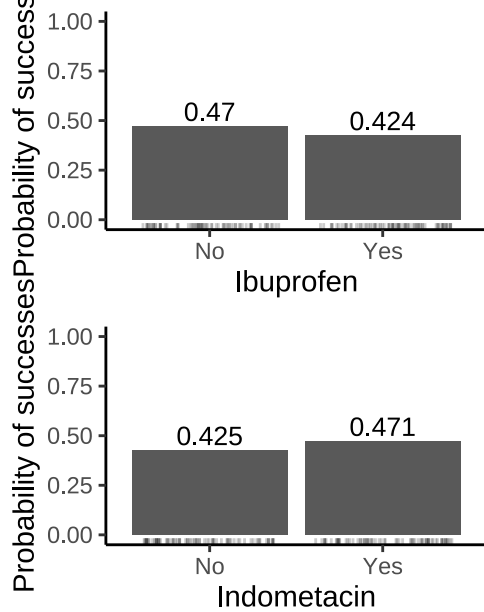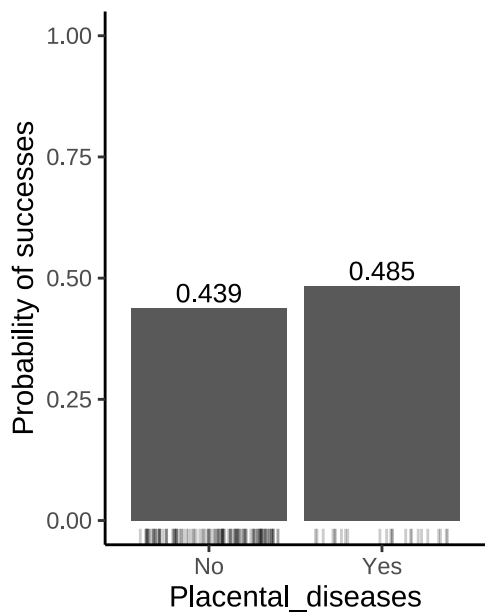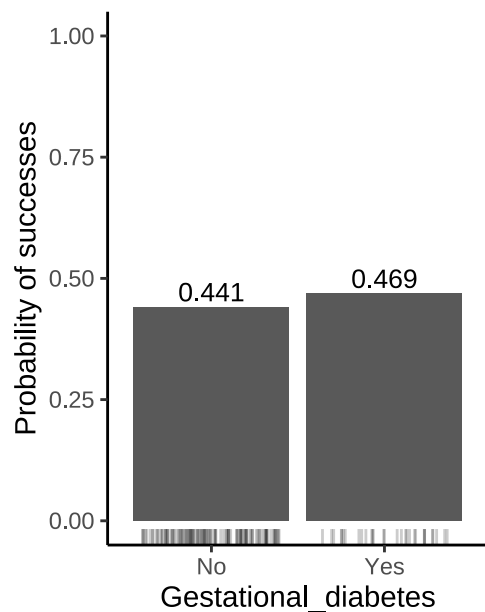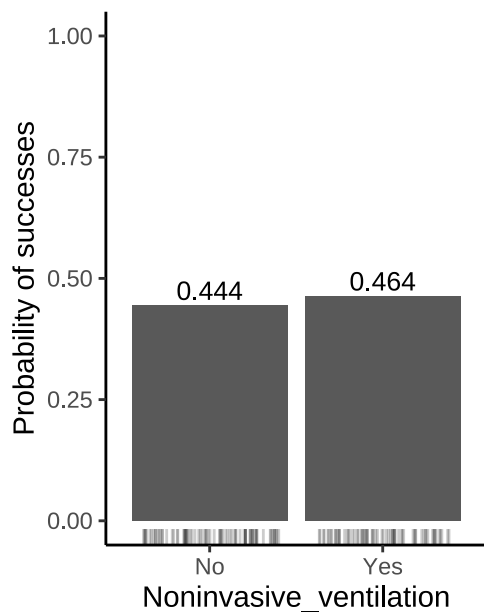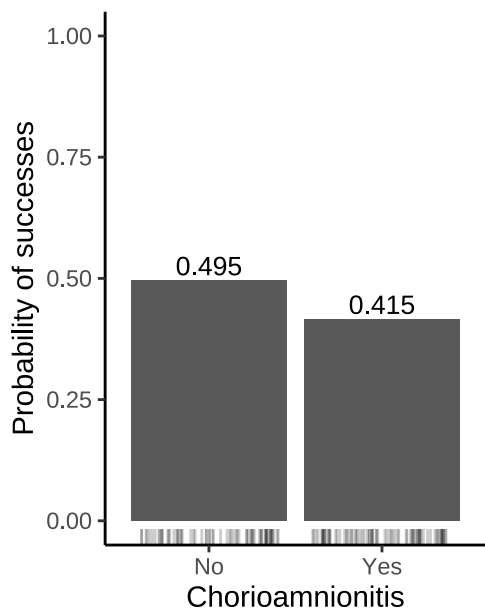

Supplement: Supplementary file 2 [file Datasheet2.pdf]
